# Civic and Citizenship Education: From Big Data to Transformative Education

**DOI:** 10.1007/978-3-030-66788-7_7

**Published:** 2021-02-27

**Authors:** Heidi Biseth, Bryony Hoskins, Lihong Huang

**Affiliations:** 19 Department of Culture, Religion and Social Studies, University of South-Eastern Norway, Drammen, Norway; 20 Department of Social Sciences, University of Roehampton, London, UK; 21 Norwegian Social Research--NOVA, Oslo Metropolitan University, Oslo, Norway; 22 Department of Culture, Religion and Social Studies, University of South-Eastern Norway, Drammen, Norway; 23 Department of Social Sciences, University of Roehampton, London, UK; 24 Norwegian Social Research—NOVA, Oslo Metropolitan University, Oslo, Norway

**Keywords:** Citizenship education, Education for sustainable development, Transformative civic education, International Civic and Citizenship Education Study (ICCS)

## Abstract

This chapter brings the results from the chapters in this book together to explore how civic and citizenship education can be or is relevant in a context beyond school. We have demonstrated that IEA’s International Civic and Citizenship Education Study (ICCS) provides results based on conventional understandings of democracy but also includes elements allowing us to address issues supporting the need for profound changes in education and, hence, relevant for both policymakers and practitioners working to make education relevant to the world the students are entering. To enable and support our young citizens in their civic actions in a rapidly changing world, we need transformative civic education. A Nordic lens on civic and citizenship education allows questions relevant for an advanced technological future and promoting civic engagement through education for environmental sustainability. How to measure and to teach civic and citizenship education is relevant to the extent that it is addressing the reality in which we live, the societal and environmental challenges we face.

## Introduction

The long history of democracy, equality, and human rights in the Nordic countries are often seen as an example, a “northern light” that many countries wish to learn from. These countries’ relatively extensive welfare systems, rather egalitarian societies, combined with the comprehensive public-school systems, stand out as a common Nordic model. This model was expected to influence civic education and young people’s civic competences. In the following sections, we elaborate on how the topics of this book have addressed elements of this Nordic model. This includes a contestation of the passive-active dichotomy in the discourse of youth, including the Nordic youth (Chapter 10.1007/978-3-030-66788-7_2), with a call for an elaboration of what counts as democratic engagement. Authors of Chapter 10.1007/978-3-030-66788-7_4 echoed this when analyzing the digital citizen and the need for schools, teachers, and researchers to capture all the ways in which Nordic youth civically engage in a fast-developing digital space. In addition, Chapter 10.1007/978-3-030-66788-7_3 addressed how principals in the Nordic countries work with citizenship education, highlighting their prioritization of critical thinking, and explaining that this is part of the educational *Bildung* ideals of the Nordic countries and a continuation of the original Nordic citizenship ideals. Despite the Nordic comprehensive school, a part of the relatively egalitarian Nordic model, Chapter 10.1007/978-3-030-66788-7_5 examined how socioeconomic inequalities affect civic competences and schools’ facilitation of civic learning. The socioeconomic background of students also influences their environmental citizenship as analyzed in Chapter 10.1007/978-3-030-66788-7_3.

The international results from the IEA ICCS 2016 study indicate a need to strengthen the capacity for civic and citizenship education that take account of all students. This can help to alleviate the performance gap between girls and boys, and between students based on socioeconomic background, migrant status, and other factors (Schulz et al. 2018). Currently, there is no obvious link between the results from large datasets, such as the ICCS study, to evidence an impact on an education system, in particular to the extent that the changes are noticeable by the students. International large-scale studies can inform the research community. Yet, so far, the ICCS studies have, it is argued, had limited influence on policymakers, teacher education, and school practices (see e.g., Biseth et al. 2021, in press). The themes in the ICCS studies are, nevertheless, of importance to stakeholders in the education sector because they provide information on civic and citizenship education practice and on the democratic knowledge, skills, and competences of the youth. Thus, more effort is needed to ensure the connection between evidence with policy and practice. In addition, as democracy is not a constant and changes rapidly, studies like ICCS need to stay up to date in order to remain relevant to policymakers.

## Nordic Lights on the Research, Policy, and Practice Triangle

Too often, complex research using ICCS data remains within academic journal articles and the results are not translated into the education field. With this book we have aimed to make some such research from the Nordic countries readily available for researchers through each thematic chapter. In this concluding chapter, we aim to sum up and identify potential implication for the education field, both for policymakers in each of the Nordic countries as well as teachers, teacher educators, and other practitioners in the education sector. To achieve these aims we explore the implications from each chapter thematically. Although each chapter touches on these matters, we want to further elaborate on how policy and practice can attempt to tackle the issues that have been raised concerning effective citizenship education, socioeconomic inequalities and learning, the interplay of power in schools and digital citizenship education, and environmental citizenship education in the Nordic countries. ICCS can form a basis for collaboration and enable the translation of research into everyday practice in schools. The most effective way for this to happen is through collaboration between the triangle of research, policy, and practice.

### Effective Practice: The Nordic Citizenship Education Model

The Nordic picture of civic education can be understood as a success story according to the results of Chapter 10.1007/978-3-030-66788-7_2, with Nordic countries having high and rising levels of civic knowledge and positive values and attitudes towards equality. The youth may lag behind other countries on participatory attitudes but some research suggests that these differences appear to be reduced as young people get older (Amnå and Zetterberg 2010). So, what then can be considered the success factors of Nordic civic education that other countries’ policy and education practice can learn from?

Chapter 10.1007/978-3-030-66788-7_3 outlines how the Nordic education model on citizenship emphasizes social mobility, equity, democratic participation, and citizenship within a comprehensive and unified school system and identifies that Nordic educators tend to prioritize independent and critical thinking in schools and conflict resolution (see also Hoskins et al. 2011). In terms of pedagogy, Chapter 10.1007/978-3-030-66788-7_5 shows us that Denmark, Norway, and Sweden are above the international mean for students reported experiences of an open classroom climate and students reported experiences of citizenship activities in the school. Altogether perhaps these factors can be considered useful foundations for teaching citizenship education or at least for the reserved citizen who will become active as needed and when they are older. The extensive use of digital tools for teaching and learning purposes, as discussed in Chapter 10.1007/978-3-030-66788-7_4, can be further explored and exploited to increase civic engagement among youth at platforms with lower thresholds for participation than conventional democratic methods, when using social media.

### Inequalities and Learning Political Engagement

Nevertheless, even in Nordic countries where comprehensive education is prioritized, there are significant socioeconomic differences in levels of civic competence (measured in Chapter 10.1007/978-3-030-66788-7_5 by civic knowledge, citizenship efficacy, and voting intentions) and student environmental citizenship (measured in Chapter 10.1007/978-3-030-66788-7_6 by student concerns, values, engagement, and intended actions towards protecting the environment). Two processes within the school were seen to influence these results: access to the learning and differential effects from the experience. First, Chapter 10.1007/978-3-030-66788-7_5 identified that there were socioeconomic inequalities in access to the learning of political engagement across all Nordic countries for both open classroom climate and citizenship activities at school. Access was found to be an issue at two levels. At the school level we found that schools that have more disadvantaged students report less experiences of these two types of activities. At the level of the students, we found that students from more advantaged backgrounds report more experiences of open classroom climate and citizenship activities at school than their less advantaged peers in the same school. Second, there were differential effects of the learning experiences for different social groups and there were some positive examples where these political learning experiences (open classroom climate and citizenship activities at school) actually benefited disadvantaged students more. From these two results we can conclude that it may well be possible to use the education system to create greater levels of equality in political engagement if disadvantaged young people are supported to access these learning opportunities.

The implication for policy and practice is that in schools with high numbers of students from a low socioeconomic background, and even within what is considered excellent comprehensive school systems in the Nordic countries, there is a need to step up efforts to organize activities that allow these students to practice democracy. The results showed that these schools trailed those with a more privileged intake in providing this important learning opportunity. Moreover, there is an important assignment for all schools to encourage students of disadvantaged backgrounds to make use of the civic learning opportunities provided (Hoskins and Janmaat 2019) as our findings from Chapter 10.1007/978-3-030-66788-7_5 reveal that students from less advantaged backgrounds within the same classroom as their more advantaged peers report participating less in citizenship activities. The ICCS data cannot provide answers as to why this is the case but it could be because these students feel less able to, they are less interested to do so, or that their teachers ask the more advantaged students to do these activities.

To tackle the inclusion of all social groups within open classroom discussion and civic activities requires changes to initial and continuing professional development of teachers and school leaders. Teacher education and local professional development programs also need to include substantive content on social class and on how to include young people from disadvantaged communities within the school and on how social class influences classroom interaction.^1^ (see e.g., Burner and Biseth 2016). Inclusive teaching involves developing a learning environment whereby all students not only have the right to access all learning activities but are in reality included within class discussions and democratic activities in the school community (Carrington et al. 2015; Biseth 2010). This requires teachers to develop a deep knowledge of their students’ backgrounds and for them to be able to analyze the reasons for difficulties in accessing democratic activities and having their say in the classroom (Ainscow et al. 2006). Such knowledge may enable teachers to rethink how to address, for example, student defiance and non-conformity within the school environment, especially when unexpected and even unwanted behaviour occurs. These moments can, at times, be reinterpreted as potential opportunities to start a discussion on how to take action and create change in the school and wider community (Hoskins and Janmaat 2019; Nolan 2011, 2018). Thus, moments of defiance could potentially become an opportunity to plant the seed for future political engagement.

To arrive at more inclusive schools, there may be a need to implement structural changes to reduce the increasing inequalities between schools and a reconsideration of policies such as Swedish education policies on school choice that reduce the comprehensive nature of the education system. In addition, it may well be beneficial to identify curricular change for teacher education by policymakers at the relevant level and where autonomy exists for teacher education at the level of the college or university so that social and economic inequalities and their effects are better understood. Furthermore, budgets can be targeted for schools with higher numbers of disadvantaged young people to support the organization of democracy activities in these schools and to encourage the most able teachers to work in them. In addition, inspections can be organized to observe and ensure that all social groups are involved in the decision-making activities of the school.

### The Interplay of Power Within Schools

A theme that developed in Chapter 10.1007/978-3-030-66788-7_5 is the issue of power. Democracy is one form of organization of power within a country. Schools can implement to a certain degree opportunity for students to take decisions through school councils but by and large, it is another system of power where typically unelected principals take the most important decisions, teachers implement the decrees of the principal, and conformity to rules by students is obtained by a combination of reward and punishment (see e.g., Børhaug 2007a, b; Biseth 2011). According to critical theorists, the rules of the school are typically those which have been developed within middle-class households and are easier to understand and apply by those who have been brought up under these conditions (Bourdieu and Passeron 1977). Defiance in this context could be understood as a demonstration of how the system of power in schools is not equal for all (Hoskins and Janmaat 2019; Nolan 2011, 2018). It is possible to question what kind of civic agency students learn within the existing power structures in school. Additionally, practitioners may want to address how they convert democratic values into civic virtues to ensure a stronger sense of democratic practices in future classrooms and schools (White 1996; Carr and Thésée 2019).

### Digital Citizenship

Chapter 10.1007/978-3-030-66788-7_4 discusses how citizenship education and digital learning are kept in different silos within the curriculum, and despite high levels of online and collaborative equipment and a high perceived level of competence in navigating online communication, the teachers avoid working with students on social media platforms. The students are learning *about* civic engagement from digital media content. However, the teachers do not facilitate civic engagement by modelling how to participate online and in social media. On top of this, the students report that they are currently not participating in or posting on social media on civic or political topics. There are significant inequalities with this situation as the less advantaged are unlikely to have the home support on how to create digital political content and if this is not covered in school the issue of socioeconomic inequalities in digital political engagement will only increase.

To tackle this, the digital world needs to be integrated into the citizenship education curricula to inform the development of digital citizenship education. This can be developed at the national policy level and incorporated into national curricular and developed within schools themselves. Again, policymakers can make budgets available for schools to develop their digital citizenship education and guide both initial and teacher education to support teachers to change their teaching approaches. The same teaching and learning principles that apply for citizenship education (Hoskins and Janmaat 2019) can be applied for digital citizenship education and the active creation of digital content is likely to be the most effective method (Bowyer and Kahne 2020). In the world of YouTube and influencers, young people currently enjoy developing their own films and this could be used for civic education purposes. Moreover, simulations of content development within closed school forums may be an alternative safer space for young people to start to learn the skills for the creation of digital content and civic engagement online. Gamification in education is an upcoming trend helping to motivate students with teaching methods close to their everyday activities (Dichev and Dicheva 2017; Kocakoyun and Ozdamli 2018), and at the same time providing teachers and teacher educators with the possibilities of roleplays, play out scenarios, and creating other learning activities conducive of stimulating civic engagement relevant for students and for the future society and labour market they will occupy.

## Active Citizenship for Crisis?

### Transformative Education and Global Citizenship

The ICCS studies provide results based on conventional understandings of democracy but also include elements allowing us to address issues supporting the need for profound changes in education and, hence, relevant for both policymakers and practitioners. Faced with a world of many political conflicts, an environmental crisis and a pandemic, more than ever, our conventional democratic values and civic engagement are put to the test. Do our schools and teachers manage to display our civic virtues, in both physical and digital classrooms, towards our young citizens with whom experiences and concerns for the future may not be shared (Biesta 2006)? To what extent are schools and teacher education prepared to address, develop, and nurture civic knowledge, attitudes, values, and skills needed by our young citizens facing an uncertain future? To enable and support our young citizens in their civic actions beyond conventional democratic activities, we need transformative civic education. Transformative education is to be understood in a broad educational context as forming an ideological nexus between liberal education, progressive education, environmental education, and education for sustainable development (Mezirow 1996; Pavlova 2013). Freire utilized the term transformation, postulating that individuals would develop a critical consciousness as transformers of the world (Freire 2000). For Freire, this conscientization was linked to individual empowerment and the transformation of reality more broadly. Transformative education is tasked with fostering transformative learning for both the teacher and the learner. Here, the role of the teacher and learner become intertwined where transformative learning for both parties in the classroom through critical reflection and dialogical methodology on issues of common interest (Taylor 2017). Transformative learning in civic and citizenship education should consider personal transformation of both the learner and the teacher, going beyond its foundation as the personal transformation of only the learner (McWhinney and Markos 2003). Taylor (2017) has correctly pointed out a learner’s potential for transformation to developments of globalization and the associated change in demography, which is increasingly providing the inter-cultural integration required for transformative dialogical encounters. This is a step further from the understanding that education promoting transformation places specific emphasis on student-centered learning, democratic education, and encouraging learner action (Kitchenham 2008). Through transformative education, both schools and teacher education need to face the same realities of uncertain futures as our young citizens do, and to educate students and teachers who jointly can become agents willing and able to make an impact (Apple 2017; Sahlberg and Brown 2017). Such transformative education in civic and citizenship education is a matter for educators. Results for the ICCS studies can provide some insights into dilemmas in need of thematizing in civic and citizenship education for global citizenship.

### Climate Change—Education for Sustainable Development

Nordic countries are ranked high in international development indices. They are among the top 10 countries of the best achievement in all 17 sustainable development goals with an average of 72 points out of 100 (Sachs et al. 2019) while they are in the top 15 countries of best achievement of environmental sustainability goals with points ranging from 77 in Norway to 81.6 in Denmark out of 100 (Wendling et al. 2018). However, these results do not necessarily mean that Nordic countries have achieved all sustainable development goals and there are considerable variations between the Nordic countries despite the distinct similarities in social, political, and education systems among them.

Whilst ranking together at the top of civic knowledge achievement in both ICCS studies, Nordic education systems show considerable difference in education for environmental sustainability both from each other and in comparison with the international averages. First, comparing to the international average, a substantially lower proportion of Nordic school principals especially in Denmark (15% in the 2009 dataset and 9% in the 2016 dataset) consider “promoting respect and safeguard the environment” as one of the three most important aims of civic and citizenship education, except in Finland where near half of their principals did so (see Table 10.1007/978-3-030-66788-7_3 of Chapter 10.1007/978-3-030-66788-7_3 in this book). Second, lower than the international average of 58% are the percentages of teachers in the Nordic schools (except 60% in Finland) that have received pre-service and in-service training on subjects related to environment and environmental sustainability. Meanwhile, near the international average of 84%, most Nordic teachers (between 77% in Denmark and 92% in Sweden) feel well prepared for teaching these subjects (Tables 10.1007/978-3-030-66788-7_2 and 10.1007/978-3-030-66788-7_6 in Schulz et al. 2018). Third, teachers of Nordic schools report considerably lower than international averages of working with their students on several actions related to environmental sustainability (Tables 10.1007/978-3-030-66788-7_6 and 10.1007/978-3-030-66788-7_6 in Schulz et al. 2018). However, combinations of school learning activities related to environmental sustainability show some significant differences between the Nordic countries (Cheah and Huang 2019).

On the other hand, Chapter 10.1007/978-3-030-66788-7_6 presents how Nordic school students show remarkable unity not only in their civic knowledge achievement but also in their environmental sustainability-related attitudes, civic engagement, and future participations. First, a substantially higher proportion of Nordic students (between 62% in Finland and 68% in Sweden) than both the international average (55%) and European average (56%) consider climate change to be the biggest threat to the world future (see Table 10.1007/978-3-030-66788-7_6). Meanwhile, the 8th European Social Survey in 2015 shows that only about 20% of the adults in the Nordic countries are worried about climate change (Poortinga et al. 2018). Second, similar with the international average, the majority of Nordic students consider “taking part in activities to protect the environment” as an important indicator of a good adult citizen (see Appendix Table 10.1007/978-3-030-66788-7_6). Third, combining several indicators of their environmental sustainability-related attitudes and behaviours, Nordic students are very similar in environmental citizenship regardless of their country of residence (see Appendix Table 10.1007/978-3-030-66788-7_6 of this book; Cheah and Huang 2019).

In all previous analyses, we notice a rather large discrepancy between the low levels of priority placed on the environment by principals for citizenship education in comparison to students’ high levels of environmental citizenship, both internationally and in the Nordic contexts. Moreover, it is surprising to see how small the effect of school education practices of environmental sustainability are on student environmental citizenship in the Nordic countries, and the effect is not significant in Sweden (Cheah and Huang 2019). In general, Nordic students appear to be ahead of their school principals and the adult population in terms of environmental sustainability and their environmental citizenship which seems to have limited dependence on education practices at their schools as well as home background such as ethnicity and parents’ higher education attainment (Cheah and Huang 2019).

This mirrors the current global youth movement, way ahead of the adult population, illustrated by the school strike for the environment initiated by the Swedish teenager Greta Thunberg who was at age 14 during the ICCS 2016 study. These results call for serious reflections among school staff and teacher educators and action on education for environmental sustainability.

### In the Wake of the COVID-19 Pandemic

We dare to venture into a topic not touched upon in any of the previous chapters, but a contemporary situation significantly influencing our lives and highly relevant to civic and citizenship education: The COVID-19 pandemic developed while this book was written (Worldometers 2020). This situation illustrates how the world is interconnected and the need to direct our attention and efforts into developing global citizenship education. COVID-19 spread rapidly in late 2019 from China to all continents. Populations have been instructed on a large scale to clean hands and keep a safe distance from those outside of your household. With quarantine regulations and restrictions on being outdoors, grassroot initiatives emerged among citizens supporting those in need of help (grocery shopping, buying medicine and other necessities).

While COVID-19 has spread, local and world travel has slowed down, and air pollution has dropped. This rapid change in our behaviour has resulted in a climate benefit, an unintended result of the politically decided lockdown, yet good for our planet. For the Nordic students already understanding environmental sustainability-related attitudes and engagement related to democratic citizenship, the COVID-19 pandemic may ensure a current and future forceful engagement. For practitioners, this situation may illustrate the need to put more emphasis on sustainable development as part of civic and citizenship education.

For those children and youth affected by the school lockdown in the Nordic countries, teaching and learning activities have been going on through digital platforms and tools. Several publishing houses have made teaching material digitally available for free during the lockdown. Teachers and students have experienced a steep learning curve in the use of digital tools and social media for learning purposes. Adding independent and critical thinking skills, and action, to their democratic capabilities, it would be possible to see digitally competent students use their competencies in a global crisis to provide critical and innovative resources (Carr et al. 2018). One example is the 17-year-old boy in Seattle, Avi Schiffmann, who has made a website, https://ncov2019.live/data, collating data from several official sources within many countries affected by COVID-19 and providing immediate updates and data to reporters and others who want critical input on a global scale (Democracy Now! 2020). In one way, it is possible to claim that Mr. Shiffmann has practiced global citizenship, making use of his innovative skills to provide a free tool in a situation of crisis.

Despite the crisis emerging due to COVID-19, youth may learn civic engagement locally based on a global situation—for example, how to help and support neighbours in need, and effective measures in everyday lives to ensure that health workers can do their job to the benefit of society. These are examples of how democratic values such as equality and solidarity, human dignity, shared responsibility, trust, respect, and compassion are, or can be, converted into practice (White 1996). During a time of crisis, youth can learn global citizenship through their everyday lives—and educators may use this in school to strengthen their work on education for sustainable development, sustainable lifestyles, and global citizenship when responding to the UN’s Sustainable Development Goal 4.7 (United Nations 2020).

What will happen with the solidarity of a global citizen when an effective vaccine for COVID-19 is developed? Will the youth experience Nordic and European politicians call for solidarity with low-income countries with poorly developed health care systems? How will the education system respond to the civic and democratic challenges in the wake of the COVID-19 pandemic? Will we see a need for transformative education preparing youth to understand how local actions may have global impacts—and their role as global citizens to join forces in critically questioning and acting upon the status quo of a world with large inequalities? This pandemic, as devastating as it is, provides youth with experiences that may influence their civic understanding, attitudes, and engagement.

## In Closing

All the analyses on data from ICCS 2009 and ICCS 2016 take as a point of departure the existing education acts and national curricula at the time of data collection. Denmark implemented a new Education Act (Børne- og undervisningsministeriet 2019) with subsequent changes in the national curricula from 2019. Finland presented the new national core curriculum late in 2014 (Vahtivuori-Hänninen et al. 2014; Finnish National Board of Education 2016). Norway implemented a new core curriculum as well as subject curricula from August 2020 (Utdanningsdirektoratet 2020a, b). Sweden had the curricula revised in 2019 (Skolverket 2019). The changes to the education policy documents have not been analyzed in this volume, but several of them will have a significant impact on civic and citizenship education. For example, democracy and citizenship becoming one of three crosscutting themes in Norwegian education, the comprehensive role of education for sustainable development, and transversal digital skills emphasized in all four countries. The education sector is responding to contemporary societal challenges and needs. This will certainly impact civic and citizenship education, and the results of the ICCS 2022 study. Questions in the ICCS studies measuring trends are important. However, changes in what the Nordic societies need of their citizens, as in other countries, and what skills and virtues are required to cope with a rapidly changing world, are not likely to be covered through questions based on what was a good citizen in the past. Perhaps a Nordic lens on civic and citizenship education could allow questions relevant for an advanced technological future in which adaptability to rapid societal changes is a treasured skill. Education for environmental sustainability would be another core element for civic engagement in which students can make use of their independent and critical thinking skills to act effectively at a local and global level. By doing so, the ICCS 2022 study in the Nordic countries can measure both international trends in addition to topics of societal challenges relevant to civic and citizenship education in the Nordic countries.

## Footnotes

1 Ethnicity, sexuality, gender, and disabilities, and intersectionality between these groups, are equally necessary to take into account.
